# Correspondence: Phantom phonon localization in relaxors

**DOI:** 10.1038/s41467-017-01395-6

**Published:** 2017-12-05

**Authors:** Peter M. Gehring, Dan Parshall, Leland Harriger, Chris Stock, Guangyong Xu, Xiaobing Li, Haosu Luo

**Affiliations:** 1 NIST Center for Neutron Research, National Institute of Standards and Technology, 100 Bureau Drive, Gaithersburg, MD 20899-600 USA; 2 School of Astronomy and Physics, University of Edinburgh, Edinburgh, EH9 3JZ UK; 3 Condensed Matter Physics and Materials Science Department, Brookhaven National Laboratory, Upton, New York, NY 11973 USA; 4 Shanghai Institute of Ceramics, Chinese Academy of Sciences, Shanghai, 201800 China

## Introduction

Manley et al.^1^ report the observation of an unexpected, weak, phonon mode located in energy between that of the transverse acoustic (TA) and soft transverse optic (TO) modes in the relaxor ferroelectric Pb[(Mg_1/3_Nb_2/3_)_1-*x*_Ti_*x*_]O_3_ (PMN-*x*PT) with Ti content *x* = 0.30. Referred to as the local mode (LM), the energy of this extra mode varies little with wave vector in the cubic (paraelectric) phase, and this was interpreted as evidence of phonon localization^1^. Any excitation that is localized in space must be extended in reciprocal space **Q**. To confirm this property, we performed a standard test^2^ on a PMN-*x*PT single crystal of nominally identical composition. This was done by comparing the rates at which the Bragg, TA phonon, and LM neutron scattering cross-sections decrease as the cubic crystallographic [100] axis is tilted out of the horizontal scattering plane by rotating about the orthogonal [010] axis. We find that the LM cross section varies with tilt in a manner identical to that of the Bragg peak. A local mode with short-range spatial correlations that decay exponentially will exhibit a broad Lorentzian peak as a function of tilt. Instead, we observe a narrow Gaussian peak. We conclude that the LM is not a real mode.

We performed a series of identical energy scans at a constant wave vector **Q** = (2,−0.35,0) in the cubic phase of a single crystal of PMN-0.29PT as a function of tilt angle (rotation) about the [010] axis. These data were normalized to monitor and corrected for the harmonic content of the incident beam. Following Manley et al.^1^, the TA and TO modes were fit to Lorentzian lineshapes, while the LM was fit to a Gaussian lineshape. The elastic cross-section, which is primarily incoherent in nature because the elastic coherent diffuse scattering is relatively weak in the cubic phase (and at wave vectors far from the zone center), was fit to a Gaussian lineshape. All fits included a constant, flat background.

**Fig. 1:**
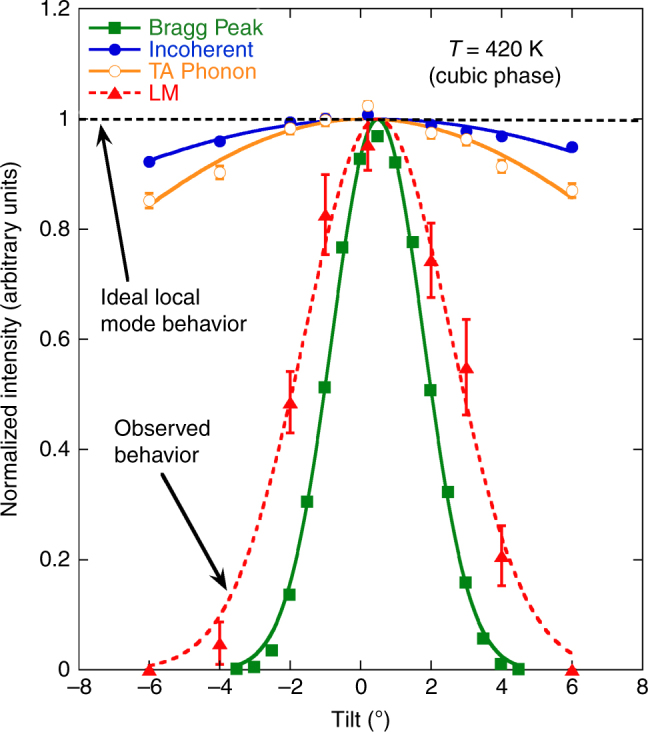
Tilt dependence of neutron scattering in cubic PMN-0.29PT. The scattering cross sections are obtained at **Q** = (2,−0.35,0). LM refers to the feature identified as a local mode by Manley et al.^1^ Lines are fits to Gaussian lineshapes. Errors bars, taken from least-squares fits, denote ±1 standard deviation

At zero tilt, we observed the LM at 11.7 meV with a linewidth of 1.8 meV (FWHM) and an energy-integrated intensity 2.7% times that of the TO mode. These values agree well with those found by Manley et al.^1^ But at tilts of ±6° the fitted LM intensities are zero: we see no evidence of the LM. To place these results in perspective, the tilt dependences of the LM and TA phonon intensities are plotted in Fig. 1 and normalized to one at zero tilt. The incoherent and the Bragg scattering cross sections are plotted too because they represent opposite extremes in terms of localization.

A truly local mode must exhibit a flat intensity tilt-profile provided the crystal remains fully illuminated by the incident neutron beam. This is exemplified by the elastic incoherent scattering cross section, which changes by <8% over the full tilt range. At the opposite extreme is the Bragg cross section, which is a delta function in both **Q** and energy; it exhibits a very narrow Gaussian dependence on tilt. The non-zero width reflects the instrumental out-of-plane wave vector resolution. Of these two extremes, both shown in Fig. 1, the LM clearly behaves like a Bragg peak. It varies with tilt far more strongly than does the TA phonon, which is obviously not a local mode. Therefore, the LM is not a local mode.

Because the LM mimics the Bragg cross section, we believe it originates from an elastic–inelastic double-scattering process, as this explains why a small tilt has such a dramatic effect on the LM intensity: tilting rotates the Ewald sphere relative to the crystal reciprocal lattice, thereby breaking the Bragg condition. This concept was used successfully by Rønnow et al.^3^ to explain the spurious “ghoston” peaks seen in CuGeO_3_. While an elastic–inelastic double-scattering process is weak, Rønnow et al. point out that even modest-sized crystals of order 1 cm^3^ can exhibit these effects. By comparison, the 200-g crystal used by Manley et al.^1^ corresponds to 25 cm^3^. In addition, phonon modes in relaxors are much broader than those in conventional perovskites such as PbTiO_3_; this fact greatly increases the chances of observing ghostons in relaxors because the double-scattering condition is more likely to be satisfied given the larger phonon energy width. Finally, the ghoston model can also explain why Manley et al.^1^ observed the LM at **Q** = (2, +0.4,0) and (2,−0.4,0): given a ghoston at (2,*q*,0) generated by Bragg scattering from **τ** = (*h*,*k*,*l*), there is always a ghoston of equal energy at (2,−*q*,0) generated by Bragg scattering from (*h*,−*k*,*l*).

Our data demonstrate that the LM scattering cross section exhibits an extremely sharp dependence on tilt that is well described by a Gaussian function. These features are inconsistent with those expected for a local mode. We conclude that the central thesis of Manley et al.^1^ is wrong.

## Methods

### Triple-axis neutron inelastic scattering

We studied an 80-g single crystal of PMN-*x*PT having nominal Ti content *x* = 0.29. The crystal was cut with {100} faces and dimensions 17.8 mm × 23 mm × 24.3 mm and oriented in the [100]–[010] scattering plane with [100] parallel to the 17.8 mm dimension. The crystal was loaded into a closed cycle ^4^He refrigerator and mounted on the NIST BT4 triple-axis spectrometer configured as in the work by Manley et al.^1^. We calibrated the BT4 wavelength and scattering angle using an alumina standard and aligned the analyzer using vanadium, which is an incoherent scatterer.

### Data availability

All relevant data are available from the corresponding author.
